# Regular endurance exercise of overloaded muscle of young and old male mice does not attenuate hypertrophy and improves fatigue resistance

**DOI:** 10.1007/s11357-020-00224-x

**Published:** 2020-07-08

**Authors:** Paul William Hendrickse, Raulas Krusnauskas, Emma Hodson-Tole, Tomas Venckunas, Hans Degens

**Affiliations:** 1 Research Centre for Musculoskeletal Science & Sports Medicine, Department of Life Sciences, Manchester Metropolitan University, John Dalton Building; Chester Street, Manchester, M1 5GD UK; 2 Institute of Sport Science and Innovations, Lithuanian Sports University, Kaunas, Lithuania; 3 University of Medicine and Pharmacy of Târgu Mureș, Târgu Mureș, Romania

**Keywords:** Hypertrophy, Oxidative capacity, Capillarization, Ageing

## Abstract

It has been observed that there is an inverse relationship between fiber size and oxidative capacity due to oxygen, ADP, and ATP diffusion limitations. We aimed to see if regular endurance exercise alongside a hypertrophic stimulus would lead to compromised adaptations to both, particularly in older animals. Here we investigated the effects of combining overload with regular endurance exercise in young (12 months) and old (26 months) male mice. The plantaris muscles of these mice were overloaded through denervation of synergists to induce hypertrophy and the mice ran on a treadmill for 30 min per day for 6 weeks. The hypertrophic response to overload was not blunted by endurance exercise, and the increase in fatigue resistance with endurance exercise was not reduced by overload. Old mice demonstrated less hypertrophy than young mice, which was associated with impaired angiogenesis and a reduction in specific tension. The data of this study suggest that combining endurance exercise and overload induces the benefits of both types of exercise without compromising adaptations to either. Additionally, the attenuated hypertrophic response to overload in old animals may be due to a diminished capacity for capillary growth.

**Electronic supplementary material:**

The online version of this article (10.1007/s11357-020-00224-x) contains supplementary material, which is available to authorized users.

## Introduction

It has long been known that mechanical loading of a muscle leads to increases in strength, muscle fiber cross-sectional area, and muscle mass, whereas regular endurance exercise induces, among other adaptations, a decrease in muscle mass, an increase in muscle oxidative capacity, and angiogenesis (Baar 2006). The combination of both hypertrophic and endurance stimuli is thought to result in a trade-off between the adaptations to each, resulting in a diminished response to the resistance or endurance exercise in comparison with these training modalities on their own (van Wessel et al. 2010). Part of such a diminished response may reflect an inverse relationship between fiber size and oxidative capacity that is thought to be due to diffusion limitations of oxygen (Degens 2012; van Wessel et al. 2010; van der Laarse et al. 1998). These predictions have however never been formally tested. Therefore, here we seek to answer the fundamental question of whether there is a trade-off using a powerful hypertrophic stimulus (denervation-induced overload) alongside regular endurance exercise. Denervation-induced overload leads to a much greater hypertrophic response than resistance exercise, but elicits otherwise similar adaptations (Alway et al. 2005).

In the capillaries, the exchange of oxygen, nutrients, heat, and metabolites with the surrounding muscle fibers takes place. In line with the potential importance of oxygen diffusion limitations for fiber size is the observation that fiber size is the main determinant of capillary supply to a fiber (Ahmed et al. 1997; Degens et al. 1994) and that hypertrophy is accompanied by angiogenesis (Degens et al. 1992, Egginton et al. 1998, Plyley, Olmstead and Noble 1998). However, in mammalian muscle, capillaries can only be placed at the periphery of a fiber. Consequently, with ongoing growth, the diffusion distance from the capillaries to the center of the fiber will increase and hence theoretically put a limit on fiber hypertrophy (Degens 2012). This may be alleviated to some extent by reducing the fiber oxidative capacity, an adaptation opposite to endurance exercise-induced adaptations. It may therefore be predicted that the hypertrophic response to overload is reduced by regular endurance exercise, and the increase in oxidative capacity induced by regular endurance exercise is diminished when accompanied by a hypertrophic stimulus.

In addition to age-related reductions in muscle strength, muscle mass, capillarization, oxidative capacity, and fatigue resistance, the adaptive responses to both hypertrophic and endurance stimuli have been shown to diminish with age in humans and rodents (Ballak et al. 2016, Petrella et al. 2006, Larsson et al. 2019, Degens and Alway 2003, Conley, Jubrias and Esselman 2000, Walters, Sweeney and Farrar 1991). The attenuated responses to overload and chronic electrical stimulation in old rodents were accompanied by a dampened angiogenic response (Ballak et al. 2016; Walters et al. 1991), and a lower capillarization in elderly people was associated with a poorer hypertrophic response (Snijders et al. 2017; Moro et al. 2019). These observations suggest that a diminished angiogenic response and/or lower capillary network in old age may underlie the diminished muscle plasticity seen within this time period (Hendrickse and Degens 2019). Given the importance of the capillary network for fatigue resistance (Tickle et al. 2020), a lower capillary density in old age (Hendrickse and Degens 2019) may also result in reduced fatigue resistance, as has particularly been seen in old age during repetitive shortening, but not isometric, contractions (Callahan and Kent-Braun 2011).

The first objective of our study was therefore to assess whether there is a trade-off between the functional and morphological adaptations to overload and regular endurance exercise. Thereto, the plantaris muscle from young and old mice was simultaneously subjected to overload by denervation of synergists and daily treadmill running. Denervation of synergists typically induces 30% hypertrophy in the overloaded muscle (Degens et al. 1992). The second objective was to assess whether muscle plasticity was (1) diminished in old age and (2) whether reduced muscle plasticity was related to a less dense microvascular network and attenuated angiogenesis. We hypothesized that (1) overload-induced hypertrophy is attenuated by endurance exercise and (2) an endurance exercise-induced increase in fatigue resistance is attenuated by overload. We also proposed that (3) both overload and endurance exercise induce angiogenesis and (4) responses to both endurance exercise and overload are blunted in old mice that is associated with a less dense microvascular network and attenuated angiogenesis.

## Materials and methods

### Animals

**Fig. 1:**
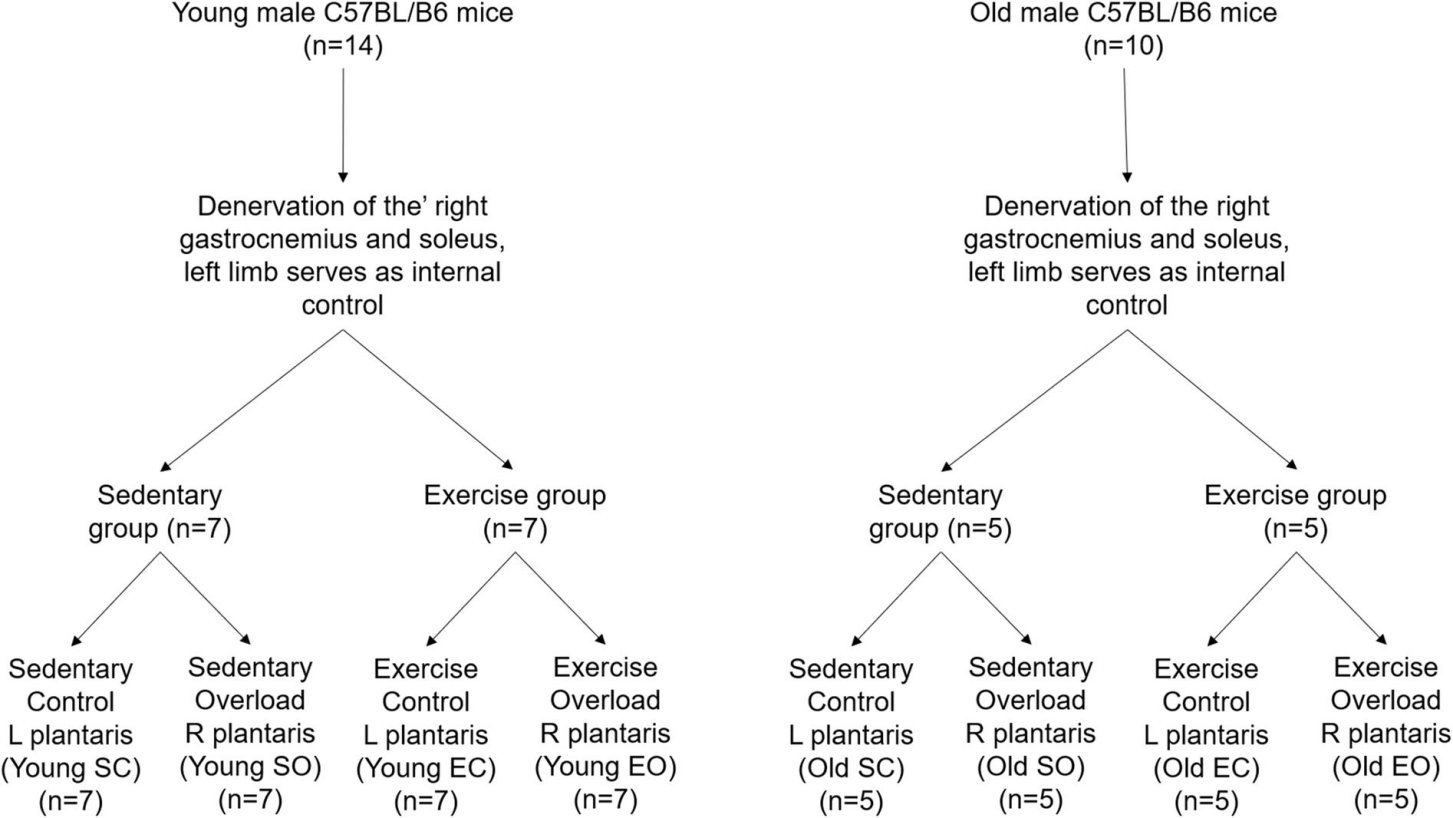
Study design. Fourteen young (12 months) and 10 old (26 months) male C57BL/B6 mice were subject to denervation surgery in which the plantaris muscles of the right hindlimb were overloaded through denervation of the gastrocnemius and soleus muscles. The left plantaris served as an internal control. Mice of each age group were then split into sedentary and exercise groups, the latter undergoing 30 min of treadmill running 5 days week^−1^. The sedentary mice had a sedentary control plantaris muscle (SC) and an overloaded plantaris muscle (SO) while the exercised mice had an exercised control plantaris muscle (EC) and an exercised overloaded plantaris muscle (EO)

Fourteen 12-month-old (young adult) and 10 26-month-old (old) male C57BL/6J mice were kept individually with ad libitum access to standard chow (LabDiet5001: protein 28.7%, carbohydrate 57.9%, fat 13.4%) and water at 22 °C on a reversed 12-h light/dark cycle at the Lithuanian Sports University. All animal procedures were approved by the Lithuanian State Food and Veterinary Service. Figure 1 outlines the study design.

### Surgery to overload the plantaris muscle

**Fig. 2:**
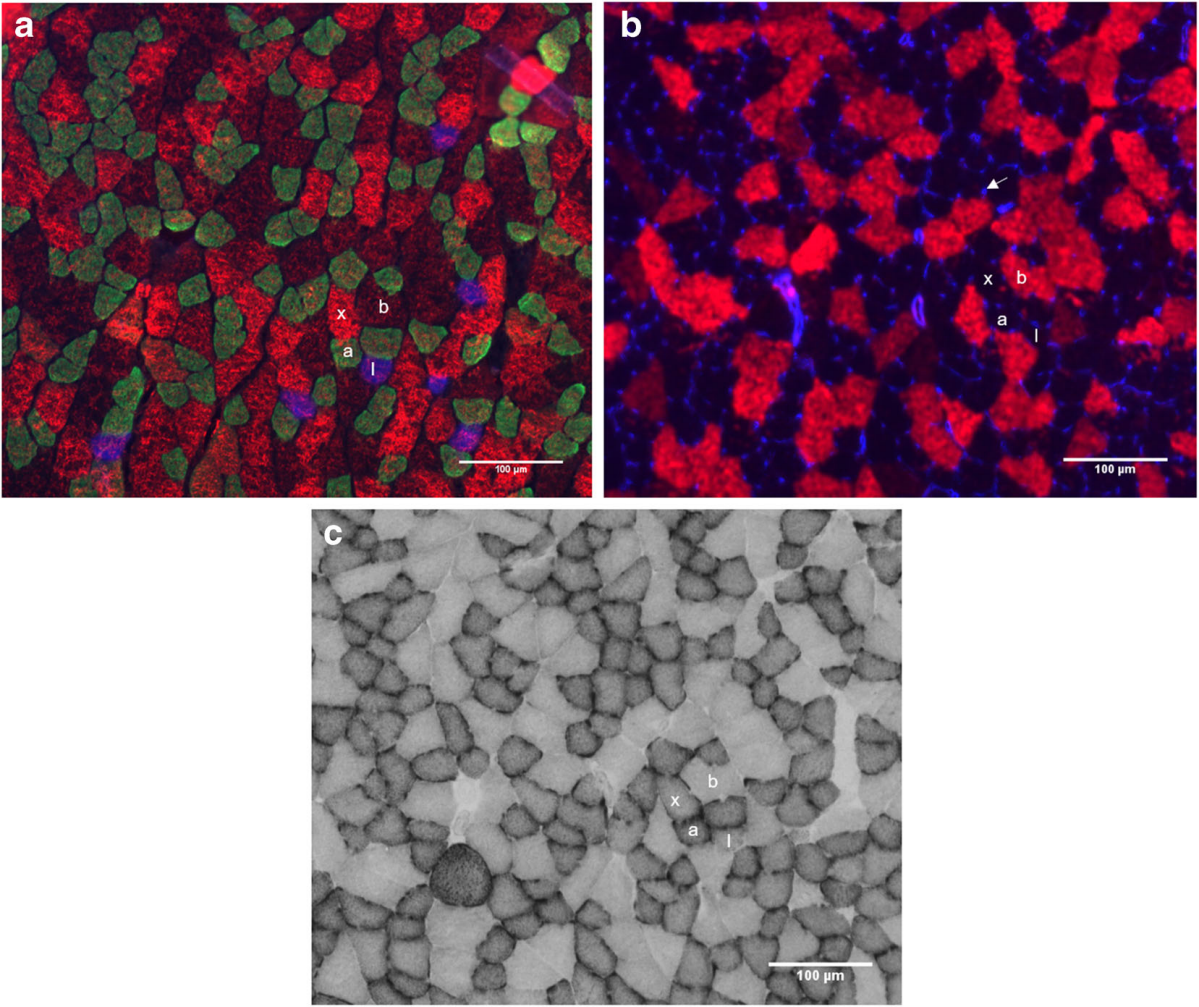
Serial sections of plantaris muscles from a young male sedentary control (SC). Sections were stained for (A) type I (blue, denoted by I), type IIa (green, by a) and type IIx fibers (red, denoted by x), (B) type IIb fibers (red, denoted by b) and capillaries (blue, denoted with an arrow), and (C) succinate dehydrogenase (SDH) activity

To overload the plantaris muscle of the right leg, the gastrocnemius and soleus muscles were denervated by cutting the medial and lateral branches of the tibial nerve as described previously (Degens and Alway 2003). A section of the nerve was removed to prevent reinnervation. The surgery was performed under aseptic conditions and animals were anesthetized with an intraperitoneal injection of a mix of ketamine (100 mg kg^−1^) (Ketamidor, Richter Pharma AG, Wels, Austria) and xylazine (16 mg kg^−1^) (Sedaxylan, Eurovet, Bladel, Netherlands) in saline. After surgery, the wound was closed with suture and the animal was given ketoprofen (2.5 mg kg^−1^) (Rifen, Eurovet, Bladel, Netherlands) as an analgesic immediately after surgery and again 24 h later. The contralateral plantaris muscle served as the internal control (Fig. 2).

### Endurance exercise

One week after recovery from surgery, half of the animals were allocated to a control group and the other half to an endurance exercise group. The endurance exercise consisted of running at a speed of 12 m min^−1^, 30 min day^−1^, 5 days week^−1^ at 0^0^ for 6 weeks. This program has been shown to induce an increase in the oxidative capacity of at least the quadriceps muscle without causing muscle damage (Savage and McPherron 2010). A shock grid was used to encourage running and mice were shocked an average of 6 times per session. Mice that refused to run during the first 5 days were removed from the exercise group and replaced with an animal from the non-exercise group. Exercise was undertaken in the dark with a red light.

### Terminal experiment

Seven weeks after the surgery to overload the plantaris muscle, the mice were anesthetized as described above. The age of the mice at the terminal experiments was 14 months and 28 months for the young-adult and old mice, respectively. The pedal withdrawal reflex was tested every 10 min and 20% of the original dose was administered to the animal when required. The gastrocnemius muscle of one leg was removed, exposing the sciatic nerve, and weighed. The animal was then moved to the in situ force measurement apparatus and its body temperature kept at 36.5 °C. The distal plantaris tendon was cut and attached to the force transducer (Dual-Mode Muscle Lever Systems 305C-LR Aurora Scientific, Ontario, Canada) using 3–0 silk suture.

The sciatic nerve was cut proximally and stimulated with incremental 0.2-ms pulses every 30 s until maximal twitch force was obtained. Then, the current was increased by 10% to supramaximally stimulate the muscle every 30 s to determine optimal length, defined as the length at which the muscle produced the maximal active twitch force.

The muscle was then stimulated twice, 5 min apart, at 200 Hz for 300 ms to determine maximal isometric tetanic force. Five minutes after the last tetanus, the muscle was subjected to a series of 330-ms 40-Hz trains once every second for 4 min to assess fatigue resistance. A fatigue index was calculated as the force in the last contraction divided by the force in the strongest contraction during the series.

The same process was then repeated on the contralateral limb. The overloaded and control muscles were stimulated in random order. After the contractile properties of the plantaris muscles were determined in both legs, the animal was euthanized with carbon dioxide. Both plantaris muscles were then removed, weighed, and mounted in OCT embedding medium in the liver (Thermo Scientific, Loughborough, UK), frozen in liquid nitrogen and stored at − 80 °C.

### Muscle morphology

Serial 10-μm sections were cut from the mid-belly of each plantaris muscle at − 20 °C using a cryostat.

Two serial slides with three sections each were blocked with 10% goat serum (Vector Laboratories, USA) in phosphate-buffered saline (PBS) for 60 min. Then, one slide was incubated for 2 h with monoclonal antibodies BA-D5 (1:600), SC-71 (1:600), and 6H1 (1:50) (Developmental Studies Hybridoma Bank, USA) in blocking solution to identify type I, IIa, and IIx fibers, respectively. The serial slide was incubated for 2 h with BF-F3 (1:100) (Developmental Studies Hybridoma Bank, USA) and biotinylated *Griffonia simplicifolia* lectin I (5 μL mL^−1^) (Vector Laboratories, USA) to identify type IIb fibers and capillaries, respectively. After three 5-min PBS washes, the first slide was incubated in secondary antibodies Alexa Fluor 350 IgG2b for type I (1:500), Alexa Fluor 488 IgG1 for type IIa (1:500), and Alexa Fluor 555 IgM for type IIx (1:500) (Thermofisher Scientific, USA). The second slide was incubated in Alexa Fluor 555 IgM for type IIb (1:500) and Streptavidin, and Alexa Fluor 350 conjugate (1:200) (Thermofisher Scientific) for capillaries. After three more 5-min washes in PBS, the slides were mounted with ProLong Diamond Antifade mountant (Thermofisher Scientific, USA).

Digital images of the deep and superficial regions of the plantaris muscle were taken using a fluorescent microscope (Zeiss Axio Imager Z1, Zeiss, Germany) at × 20 magnification. For each image, fibers were traced and typed, and coordinates of capillaries were recorded manually using BTablet (BaLoH Software, Ooij, Netherlands). Using these data, fiber cross-sectional area (FCSA), fiber type composition, capillary density (CD), capillary to fiber ratio (C:F), capillary domain size (the area geometrically supplied by a single capillary, μm^2^), local capillary to fiber ratio (LCFR) (the sum of the fractions of each domain which overlap the fiber), capillary fiber density (CFD) (LCFR divided by the cross-sectional area of a fiber, mm^−2^), and an index of the heterogeneity of capillary spacing (Log_R_SD) were calculated using AnaTis (BaLoH software, Ooij, Netherlands) (Degens et al. 1992). An average of 158 fibers were analyzed per region. The percentage of non-contractile material was calculated by subtracting the total fiber area from the total domain area and dividing by the total domain area.

### Succinate dehydrogenase

A third serial slide was stained for succinate dehydrogenase (SDH) activity as described previously (Ballak et al. 2016). The optical density (OD) of the stain at 660 nm was used as a quantitative indication of oxidative capacity (van der Laarse et al. 1989). For each sample, a series of filters with a known OD were used to create a calibration curve to adjust for variation in background staining and lighting between sections. The OD of the SDH stain was determined using ImageJ (Rasband, W.S., ImageJ, U. S. National Institutes of Health, Bethesda, MD, USA, http://imagej.net/Downloads). Integrated SDH activity was calculated as the SDH OD multiplied by the FCSA.

### Statistics

Data were analyzed with IBM SPSS version 25. Shapiro-Wilk tests were used to determine if data were normally distributed. In cases where data were not normally distributed, data were transformed logarithmically. A repeated-measures ANOVA with as within factors overload and muscle region, and between factors age, exercise, and fiber type (5 levels: IIa, IIb, IIx, IIa/IIx, IIb/IIx) was performed. The effects of overload, age, and exercise were also analyzed irrespective of fiber type on the pooled fibers. In addition to main effects, two-way interactions were considered between each factor pair. Effects and interactions were considered significant at *p* < 0.05. A Bonferroni correction was applied to test differences between fiber types.

## Results

### Body mass, muscle masses, and connective tissue

**Fig. 3:**
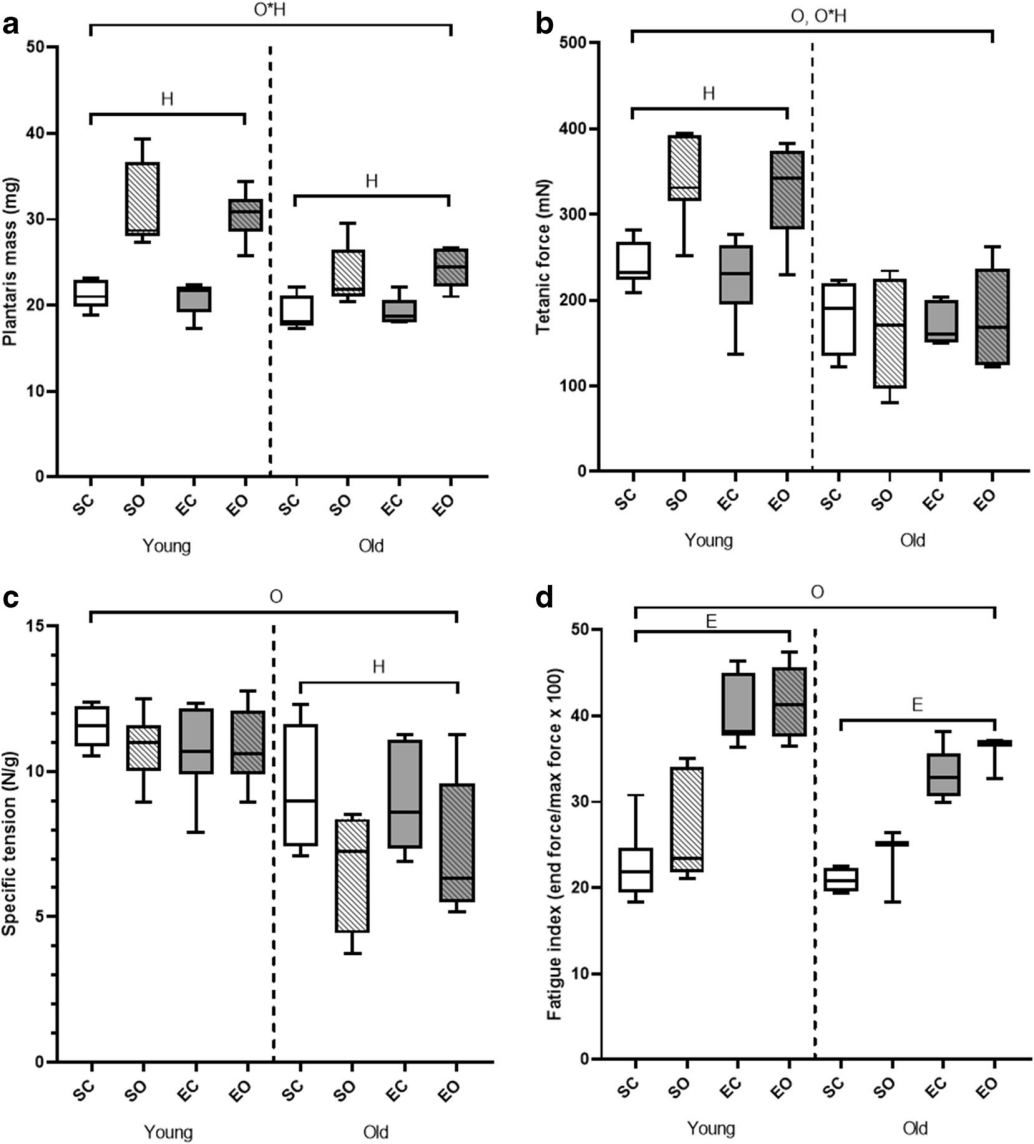
Effects of age, overload, and exercise on plantaris muscle mass and contractile properties in C57BL/6J mice. (A) Plantaris muscle mass. (B) Maximal tetanic force. (C) Specific tension as tetanic force/muscle mass. (D) Fatigue resistance. SC, sedentary control; SO, sedentary overload; EC, exercise control; EO, exercise overload. O: effect of age (*p* ≤ 0.001), H: effect of overload-induced hypertrophy (*p* ≤ 0.021), E: effect of exercise (*p* < 0.001), O*H: old age*hypertrophy interaction (*p* ≤ 0.03)

The body mass of the mice was decreased 7 weeks after induction of overload (*p* < 0.001) (Table 1). Old mice had a lower gastrocnemius muscle mass than young-adult mice (*p* < 0.001), and the denervated gastrocnemius muscle mass was lower compared with control muscles (*p* < 0.001; Table 1). The percentage of plantaris muscle cross-sectional area consisting of non-contractile material was similar in both age groups and unchanged by overload and exercise (Table 1). Control plantaris muscle mass was similar in both age groups. Overload led to an increase in plantaris muscle mass (*p* < 0.001), but the “age*overload” interaction (*p* = 0.03) was reflected by a smaller increase in mass in the old than young-adult mice (Fig. 3A). There was no significant “exercise*overload” interaction (*p* = 0.998), indicating that the overload-induced increase in plantaris muscle mass was similar in trained and non-trained animals. Similarly, plantaris mass normalized to body mass was increased by overload (*p* < 0.001) and this increase was smaller in old mice (*p* = 0.003) (Table 1).

**Table 1:** Body mass, gastrocnemius muscle mass, plantaris mass normalized to body mass, and proportion of non-contractile material in young and old C57BL/6J mice after denervation to induce hypertrophy of the plantaris muscle and/or endurance exercise

	Young (12 months)				Old (26 months)				Significant effects (*p* < 0.05)
	Sedentary pre	Sedentary post	Exercise pre	Exercise post	Sedentary pre	Sedentary post	Exercise pre	Exercise post	
Body mass (g)	38.1 ± 3.2	37.5 ± 4.8	41.5 ± 7.3	34.8 ± 5.8	41.8 ± 12.3	36.9 ± 9.4	30.6 ± 9.0	27.9 ± 8.7	T***
	SC	SO	EC	EO	SC	SO	EC	EO	
Gastrocnemius mass (mg)	129 ± 11	74 ± 11	133 ± 15	74 ± 10	122 ± 13	60 ± 7	90 ± 43	63 ± 15	O***, H***
Plantaris mass normalized to body mass (mg g^−1^)	0.572 ± 0.114	0.849 ± 0.166	0.610 ± 0.097	0.882 ± 0.105	0.548 ± 0.170	0.678 ± 0.258	0.646 ± 0.056	0.822 ± 0.112	O***, OH**
Non-contractile material (%)	8.34 ± 4.93	7.63 ± 8.22	7.33 ± 4.41	8.22 ± 2.70	10.03 ± 7.36	7.79 ± 4.93	6.98 ± 3.94	10.05 ± 9.76	

*SC*, sedentary control; *SO*, sedentary overload; *EC*, exercise control; *EO*, exercise overload. Data are expressed as means ± standard deviation. T: time effect O: age effect, H: overload effect. ** denotes *p* < 0.01 ***denotes *p* < 0.001

### Contractile properties and fatigue resistance

The maximal isometric tetanic force of the plantaris muscles was lower in old than young-adult mice (*p* < 0.001, Fig. 3B). It can be seen in Fig. 3B that the age*overload interaction (*p* < 0.001) was reflected by a higher tetanic force of the overloaded than control muscles in young animals only (*p* = 0.002). Specific tension was lower in old than young-adult animals (*p* < 0.001, Fig. 3C), and while the specific tension was maintained with overload in young animals, it was lower in overloaded muscles of old animals (age*overload interaction *p* = 0.045). Fatigue resistance was lower in old animals (*p* = 0.001), and the plantaris muscle of exercised mice had a greater fatigue resistance than those from non-exercised mice, irrespective of age or overload (*p* < 0.001, Fig. 3D).

### Fiber type composition

Since the proportion of type I and I/IIa fibers was < 1% in almost all muscle regions (Table 2), these fiber types were excluded from further analysis. The percentage of type IIa, IIa/IIx, and IIb/IIx fibers was larger in overloaded compared with control muscles (*p* ≤ 0.020) and the proportion of type IIb fibers was lower (*p* < 0.001). The proportion of type IIx fibers was lower in muscles of exercised mice (*p* = 0.037). The overload*exercise interaction (*p* = 0.039) for the proportion of type IIb/IIx fibers was reflected by a lower proportion of type IIb/IIx in control exercised, but not in overloaded exercised, than in corresponding non-exercised muscles.

**Table 2:** Fiber type composition (%) and fiber cross-sectional area (FCSA) (μm^2^) for different fiber types in the oxidative and glycolytic regions of plantaris muscles exposed to overload and endurance exercise in young and old C57BL/6J mice

	Young				Old				
	SC	SO	EC	EO	SC	SO	EC	EO	Effects and interactions
Fiber type composition (%)									
Oxidative region									
Type I	0.1 ± 0.2	6.7 ± 4.8	0.2 ± 0.5	3.2 ± 4.4	0.3 ± 0.6	0.3 ± 0.6	0.6 ± 0.8	0.2 ± 0.5	
Type I/IIa	-	0.9 ± 1.1	-	0.1 ± 0.3	-	0.2 ± 0.4	-	-	
Type IIa	30.5 ± 9.3	32.4 ± 15.3	22.2 ± 17.8	40.1 ± 10.4	19.9 ± 9.4	37.8 ± 9.5	29.3 ± 11.9	44.9 ± 10.6	R***, H***
Type IIa/IIx	8.1 ± 6.0	10.0 ± 6.8	2.1 ± 1.8	12.3 ± 5.7	9.2 ± 7.2	12.6 ± 6.8	7.6 ± 2.0	12.1 ± 3.2	R***, H**, OR*
Type IIx	22.1 ± 7.2	18.0 ± 6.5	20.8 ± 7.3	14.6 ± 4.2	19.7 ± 6.5	25.1 ± 8.5	14.9 ± 3.3	17.4 ± 10.9	E*
Type IIb/IIx	3.8 ± 2.7	7.4 ± 6.5	2.6 ± 1.5	6.4 ± 5.5	9.4 ± 8.2	4.8 ± 2.4	4.5 ± 2.6	8.6 ± 8.2	R*, H*, OH*
Type IIb	35.4 ± 9.4	24.7 ± 12.0	52.2 ± 22.8	23.4 ± 6.8	41.4 ± 18.3	19.2 ± 8.6	43.1 ± 13.9	16.9 ± 17.5	R***, H***
Glycolytic region									
Type I	-	3.3 ± 3.7	0.5 ± 1.0	0.5 ± 0.9	-	-	-	-	
Type I/IIa	-	1.3 ± 1.1	-	-	-	-	-	-	
Type IIa	7.4 ± 3.7	23.9 ± 14.1	12.9 ± 16.2	26.2 ± 7.1	14.4 ± 10.1	16.2 ± 11.8	18.1 ± 8.7	27.6 ± 8.5	R***, H***
Type IIa/IIx	6.0 ± 4.1	8.3 ± 5.4	2.2 ± 2.0	8.8 ± 8.7	3.0 ± 2.1	6.3 ± 6.7	4.4 ± 1.7	10.2 ± 6.0	R***, H**, OR*
Type IIx	14.3 ± 5.7	20.7 ± 6.2	16.6 ± 9.6	16.2 ± 10.4	19.1 ± 8.6	24.4 ± 4.2	16.1 ± 4.2	15.0 ± 10.1	E*
Type IIb/IIx	5.4 ± 3.8	10.5 ± 5.8	3.0 ± 3.2	7.0 ± 7.4	13.6 ± 9.7	12.0 ± 7.6	4.2 ± 1.5	11.5 ± 3.7	R*, H*, OH*
Type IIb	67.0 ± 10.8	32.0 ± 12.5	64.8 ± 25.8	41.3 ± 9.9	49.3 ± 18.4	41.1 ± 14.4	57.1 ± 10.1	35.6 ± 13.7	R***, H***
FCSA (μm^2^)									
Oxidative region									
Type I	367 ± 127	1057 ± 205	706 ± 140	940 ± 329	619 ± 76	735 ± 23	617 ± 315	538 ± 12	
Type I/IIa	-	1102 ± 39	-	1364 ± 20	-	750 ± 59	-	-	
Type IIa	709 ± 195	1362 ± 364	654 ± 192	1237 ± 454	618 ± 151	1075 ± 262	533 ± 157	1092 ± 298	O**, R***, H*** b***, x***, ax***, bx***
Type IIa/IIx	1124 ± 193	1784 ± 357	1146 ± 192	1722 ± 571	805 ± 257	1506 ± 273	745 ± 221	1416 ± 375	O*, H*** a***, b***, x**, bx***
Type IIx	1283 ± 290	2109 ± 420	1121 ± 257	2090 ± 426	1021 ± 271	1742 ± 370	946 ± 252	1648 ± 469	O*, R***, H*** a***, b***,ax**, bx*
Type IIb/IIx	1577 ± 238	2429 ± 705	1454 ± 217	2199 ± 519	1127 ± 312	1687 ± 274	1221 ± 226	1767 ± 386	O***, H***, OH**, RE*, HE*** a***, x* ax***
Type IIb	1667 ± 285^a^	2809 ± 706^a^	1651 ± 388^a^	2566 ± 529	1344 ± 351	1863 ± 433	1228 ± 334	2071 ± 471	O***, H*** a***, x***, ax***
Glycolytic region									
Type I	-	1102 ± 147	6900 ± 69	814 ± 27	-	-	-	-	
Type I/IIa	-	1177 ± 191	-	-	-	-	-	-	
Type IIa	647 ± 123	1456 ± 509	574 ± 133	1011 ± 315	521 ± 126	895 ± 178	497 ± 145	773 ± 210	O**, R***, H*** b***, x***, ax***, bx***
Type IIa/IIx	867 ± 187	1855 ± 395	837 ± 134	1532 ± 238	663 ± 273	1506 ± 273	674 ± 102	1232 ± 272	O*, H*** a***, b***, x**, bx***
Type IIx	1065 ± 282	2253 ± 524	927 ± 195	1838 ± 356	844 ± 285	1401 ± 452	850 ± 196	1552 ± 437	O*, R***, H*** a***, b***,ax**, bx*
Type IIb/IIx	1327 ± 244	2906 ± 714	1366 ± 396	1871 ± 376	1006 ± 267	1769 ± 302	1232 ± 272	1679 ± 419	O***, H***, OH**, RE*, HE*** a***, x* ax***
Type IIb	1709 ± 370	3136 ± 631	1528 ± 338	2378 ± 579	1242 ± 322	1978 ± 473	1142 ± 266	1990 ± 475	O***, H*** a***, x***, ax***

*SC*, sedentary control; *SO*, sedentary overload; *EC*, exercise control; *EO*, exercise overload. Data are expressed as means ± standard deviation. O: age effect, R: region effect, H: hypertrophy effect, E: exercise effect, OR: age*region interaction, OH: age*hypertrophy interaction, OE: age*exercise interaction, RH: region*hypertrophy interaction, RE: region*exercise interaction, HE: hypertrophy*exercise interaction, a: significantly different from type IIa, b: significantly different from type IIb, x: significantly different from IIx, ax: significantly different from type IIa/IIx hybrid, bx: significantly different from type IIb/IIx hybrid, *p < 0.05, **p < 0.01, ***p < 0.001

The proportions of type IIa and IIa/IIx fibers were greater in the oxidative than in the glycolytic region of the plantaris muscle (*p* < 0.001, Table 2), while for type IIb and IIb/IIx fibers, the opposite was found (*p* ≤ 0.029). The higher proportion of type IIa/IIx fibers in the oxidative region when compared with the superficial region was more pronounced in muscles from old than from young-adult mice (age*region interaction *p* = 0.047).

### Fiber cross-sectional area

**Fig. 4:**
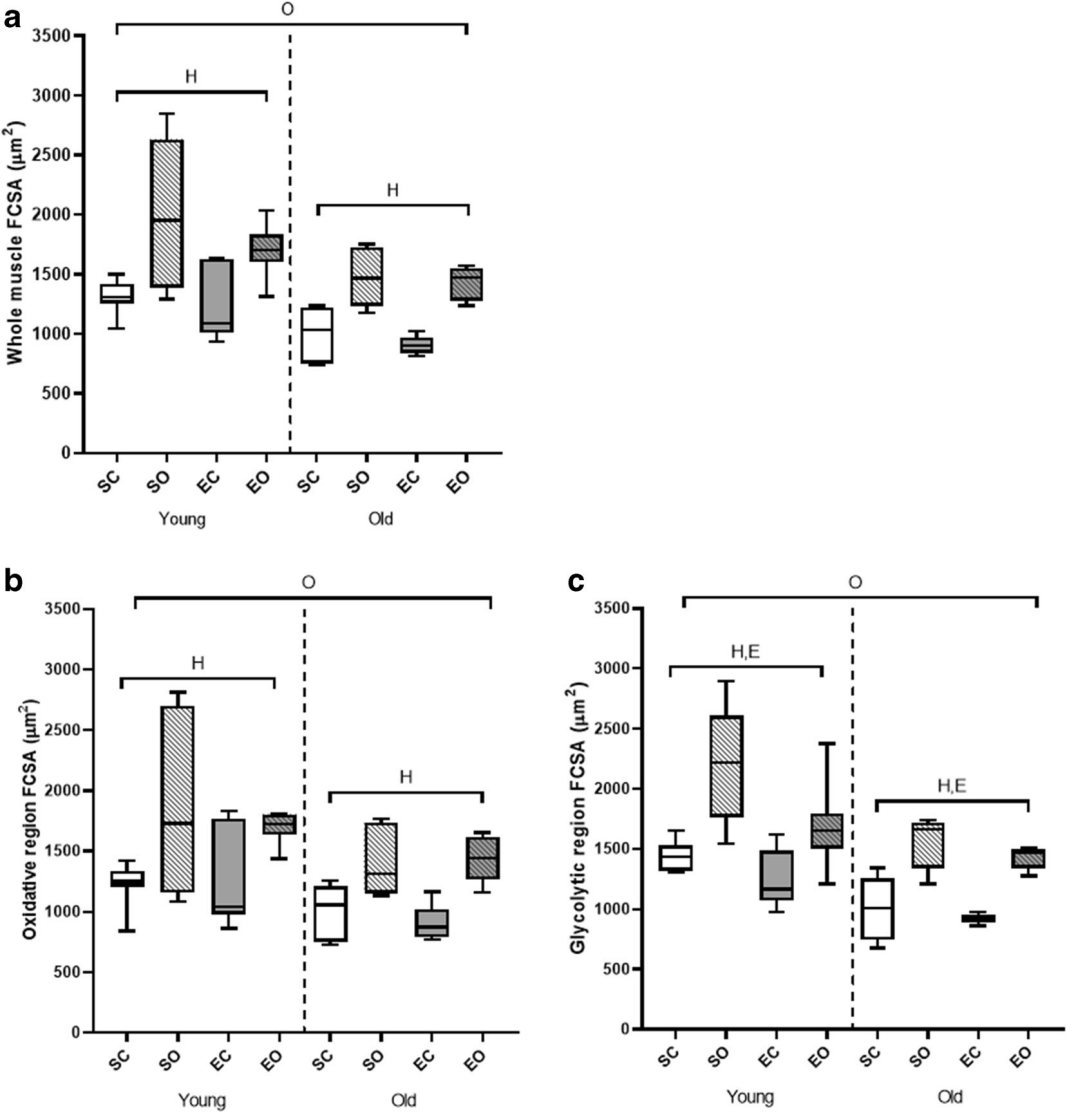
Effects of age, overload, and exercise on fiber cross-sectional area (FCSA) C57BL/6J mice. (A) Whole muscle. (B) Oxidative region. (C) Glycolytic region. SC, sedentary control; SO, sedentary overload; EC, exercise control; EO, exercise overload. O: effect of age (*p* < 0.001), H: effect of overload-induced hypertrophy (*p* < 0.001), E: effect of exercise (*p* = 0.019)

The pooled FCSA was lower in the plantaris muscles of old than young animals (*p* < 0.001, Fig. 4A) and was increased with overload in both age groups (*p* < 0.001).

The pooled FCSA was smaller in the oxidative than the glycolytic region (*p* < 0.014, Fig. 4 B and C). Overload led to an increase in average FCSA in both regions of the muscle (*p* < 0.001). Exercised mice had a lower pooled FCSA in the glycolytic, but not the oxidative, region of the muscle (*p* = 0.019) (region*exercise effect *p* = 0.017, Fig. 4 B and C).

We also studied differences between fiber types, region, age, overload, and exercise (Table 2). As with fiber type composition, type I and I/IIa fibers were excluded from statistical analyses due to their low prevalence in the plantaris muscle. Type IIa fibers were smaller than fibers of all other types (*p* < 0.001), and type IIb and IIb/IIx were larger than fibers of all other types (*p* ≤ 0.044). Type IIa and IIx fibers were larger in the oxidative than in the glycolytic region (*p* < 0.001).

While the FCSA was greater in overloaded muscles irrespective of fiber type (*p* < 0.001), the hypertrophic response was less in type IIb/IIx fibers in old than young-adult muscles (age*hypertrophy interaction *p* = 0.007). Exercise resulted in a decrease in the FCSA of type IIb/IIx fibers in the glycolytic but not in the oxidative region of the muscle (region*exercise interaction *p* = 0.045) and attenuated the hypertrophic response of type IIb/IIx fibers (overload*exercise interaction *p* < 0.001).

### Overall capillarization

**Fig. 5:**
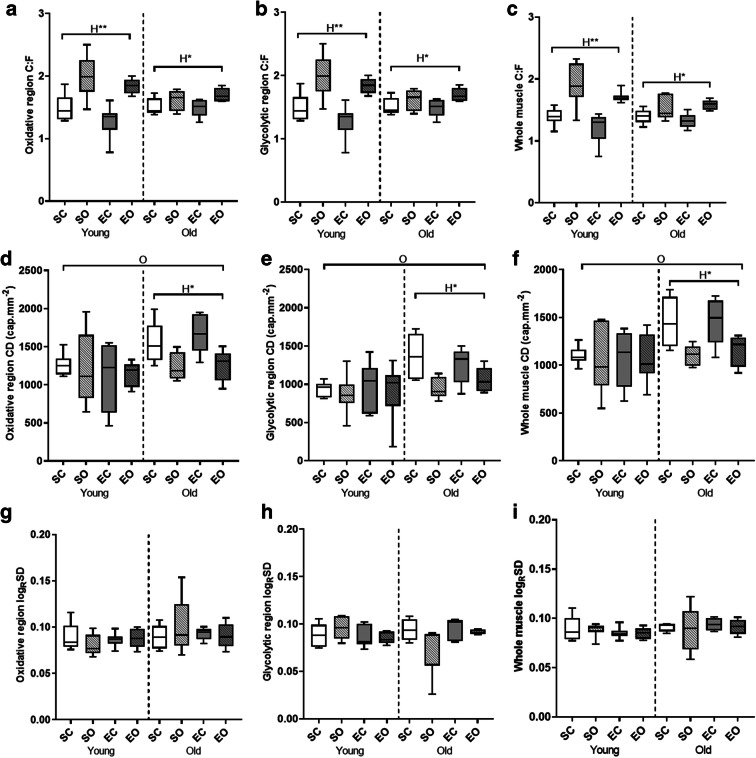
Effects of age, overload, and exercise on skeletal muscle capillarization in C57BL/6J mice. (A), (B), and (C) show capillary to fiber ratio (C:F) in the oxidative region (A), glycolytic region (B), and whole plantaris muscle (C). (D), (E), and (F) show capillary density (CD) in the oxidative region (D), glycolytic region (E), and the whole muscle (F). (G), (H), and (I) show the logarithmic standard deviation of the radius of the capillary domains (LogRSD) in the oxidative region (G), glycolytic region (H), and whole muscle (I). SC, sedentary control; SO, sedentary overload; EC, exercise control; EO, exercise overload. O: effect of age (*p* < 0.001), H*: effect of overload-induced hypertrophy (*p* < 0.005), H**: effect of overload-induced hypertrophy (*p* < 0.001)

The oxidative region of plantaris muscles had greater C:F than the glycolytic region (Fig. 5 Aand B; *p* < 0.001). Overload increased the C:F in the plantaris muscle (*p* < 0.001, Fig. 5A–C), but less so in old than young-adult mice (age*overload interaction *p* = 0.010). There was no significant effect of the exercise program on C:F (Fig. 5A–C).

The oxidative region of the plantaris muscle had a higher CD compared with the glycolytic region (*p* < 0.001, Fig. 5 D and E). The plantaris muscles of old animals had a higher CD than young animals (*p* < 0.001, Fig. 5D–F). Overloaded muscles demonstrated a lower CD compared with control muscles in old (*p* = 0.005) but not in young animals (age*hypertrophy interaction *p* = 0.028).

The heterogeneity of capillary spacing (Log_R_SD) was not significantly affected by region, age, overload, or exercise (Fig. 5G–I).

### Local capillary to fiber ratio

Supplementary Table 1 shows the LCFR data. Each fiber type had a greater LCFR in the oxidative than in the glycolytic region (*p* ≤ 0.044) except for type IIa/IIx fibers. The LCFR of type IIb fibers from old muscles was lower than that in young-adult animals (*p* = 0.019). The overload-induced increase in LCFR in all fiber types was less in old than young animals (age*hypertrophy interaction *p* ≤ 0.030), except for type IIa/IIx fibers that demonstrated a similar increase in both age groups. The age*exercise interaction (*p* ≤ 0.033) for type IIb and IIb/x fibers was reflected by an exercise-induced decrease in the LCFR of these fibers in young-adult, and an increase in old animals (Supplementary Table 1).

### Capillary fiber density

In Supplementary Table 1, it can be seen that for all fiber types, except type IIb/IIx fibers, the CFD was greater in the oxidative than in the glycolytic region of the plantaris muscle (*p* ≤ 0.013). The CFD was higher in old than young-adult animals, irrespective of fiber type (*p* ≤ 0.042). Old animals demonstrated greater differences in type IIa/IIx CFD between regions than young animals (age*region interaction *p* = 0.004).

Overloaded muscles had a lower CFD for all fiber types than control muscles (*p* ≤ 0.008), with greater reductions in type IIa and IIa/IIx fibers of old than young-adult mice (age*overload interaction *p* ≤ 0.003). The reduction in CFD of type IIa/IIx fibers after overload was less in the glycolytic than oxidative region of the muscle (region*hypertrophy interaction *p* < 0.001). There was also a region*exercise interaction (*p* ≤ 0.047) for type IIa/IIx and IIb/IIx fibers.

### Succinate dehydrogenase

**Fig. 6:**
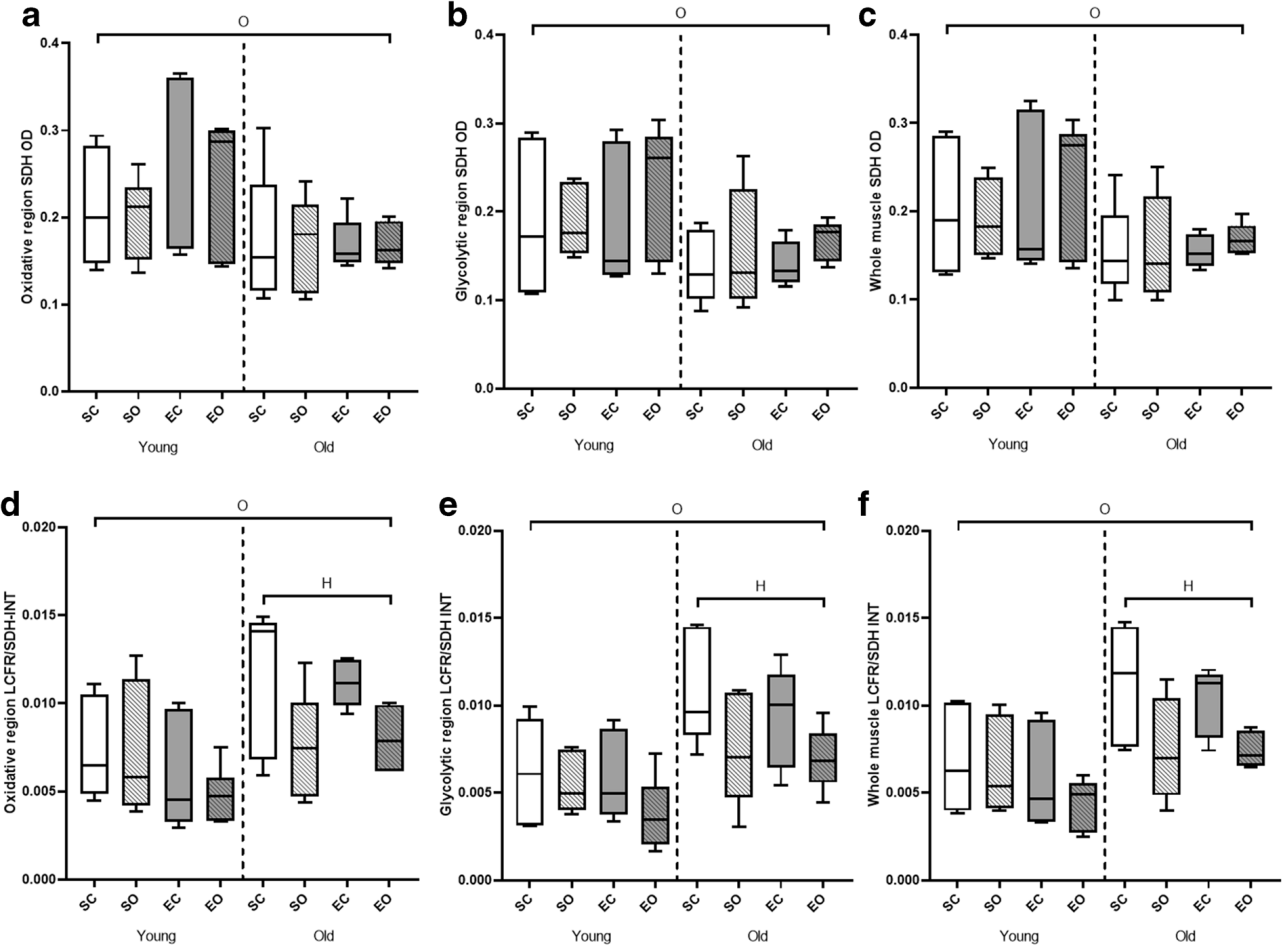
Effects of age, overload, and exercise on succinate dehydrogenase optical density (SDH OD) and local capillary to fiber ratio (LCFR):integrated succinate dehydrogenase activity (SDH-INT) in C57BL/6J mice. (A), (B), and (C) show SDH OD in the oxidative region (A), glycolytic region (B), and whole plantaris muscle (C). (D), (E), and (F) show LCFR/SDH-INT in the oxidative region (D), glycolytic region (E), and the whole muscle (F). SC, sedentary control; SO, sedentary overload; EC, exercise control, EO, exercise overload. O: effect of age (*p* ≤ 0.038), H: effect of overload-induced hypertrophy (*p* < 0.001)

Old animals demonstrated lower average SDH OD than young-adult animals (*p* < 0.001, Fig. 6A–C), which was the case for each fiber type, except type IIb fibers (*p* ≤ 0.033, Supplementary Table 1). The SDH OD of type IIa and type IIa/IIx fibers was lower in overloaded muscle (*p* ≤ 0.010), but this overload-induced reduction was less pronounced in old animals (age*hypertrophy interaction *p* = 0.008). Additionally, the type IIa/IIx fibers of young animals showed an increase in SDH OD with exercise, whereas old animals did not (age*exercise interaction *p* = 0.020). The region*overload (*p* = 0.004) and region*exercise (*p* = 0.013) interactions for the SDH OD of type IIb/IIx fibers were difficult to interpret as there were no consistent patterns visible.

### Supply:demand ratio

To assess differences in the matching of oxygen supply (LCFR) to demand (SDH-INT) of a fiber, the LCFR/SDH-INT was calculated as a measure of the supply:demand ratio (Fig. 6D–F). Old mice had a greater LCFR/SDH-INT than young-adult mice (*p* = 0.038, Fig. 6A–C). The age*overload interaction (*p* = 0.033) was reflected by a reduction in LCFR/SDH-INT in old (*p* < 0.001) but not young-adult animals. There were also region*overload (*p* = 0.003) and region*exercise (*p* = 0.014) interactions.

## Discussion

The main observations of the present study are that in both age groups, overload-induced hypertrophy was not blunted by regular endurance exercise and regular endurance exercise-induced increases in fatigue resistance were not impaired by overload. The attenuated hypertrophic response to overload in old mice was associated with a reduced specific tension and impaired angiogenesis. These data indicate that (1) combination of endurance exercise and overload elicit the benefits of both types of exercise without compromising specific adaptations and (2) the attenuated hypertrophic response in old age may be attributable to impaired angiogenesis.

### Effects of ageing on muscle function and morphology

Ageing has detrimental effects on skeletal muscle morphology, plasticity, and function (Larsson et al. 2019). Many studies have used rodents to investigate the mechanisms of musculoskeletal ageing and they are indeed useful models for human muscle ageing, provided that the correct age groups are chosen (Ballak et al. 2014b). For instance, we found an age-related decrease in fatigue resistance in 28-month-old mice, while in a previous study using 25-month-old mice, similar to early ageing (60–70 years) in humans (Ballak et al. 2014b), no significant decrease in fatigue resistance was found (Ballak et al. 2016). The age-related decrement in fatigue resistance may largely be due to the lower oxidative capacity found in the old animals, something also seen in elderly humans (Conley et al. 2000; Proctor et al. 1995), as well as to impaired blood flow (Irion et al. 1987).

In this study, we found a lower type II FCSA in the old mice (type I fibers were too few in the plantaris muscle), similar to the age-related type II atrophy observed in humans (Lexell et al. 1988; Coggan et al. 1992). Although this was not seen in 25-month-old mice (Ballak et al. 2014a), the specific tension was already lower at that age, which is in agreement with the reduced specific force found in humans from the 6th decade of life (early ageing) (Ballak et al. 2014a; Jubrias et al. 1997). While CD was greater in the old mice studied here, this is likely due to the lower FCSA in old muscle (Larsson et al. 2019), as indeed reflected by the similar C:F in the non-overloaded (control) plantaris muscles from young and old animals. The decrease in LCFR for type IIb fibers with age found here is comparable with the reduction in type II fiber capillarization seen in the muscle of elderly humans (Proctor et al. 1995; Nederveen et al. 2016).

### Effects of regular endurance exercise

In the present study, we showed that endurance exercise induced an improved fatigue resistance. While fatigue resistance is positively related to the density of capillary network (Tickle et al. 2020) and oxidative capacity (Burke et al. 1973), there was no concomitant increase in capillarization or oxidative capacity. It has been shown that slower myosin isoforms are more efficient during isometric contractions (Stienen et al. 1996) and therefore, the reduction in the proportion of type IIx fibers with endurance exercise may contribute to greater fatigue resistance during repeated isometric contractions. In addition, improvements in vasodilatory function and maximal blood flow after regular endurance exercise (Snell et al. 1987) may contribute to the improved muscle performance.

The absence of endurance exercise-induced increases in C:F, CD, or SDH OD was unexpected, as this exercise program has been reported to induce endurance exercise adaptations in muscles from C57BL/6J mice in a previous study (Savage and McPherron 2010). The discrepancy between their work and our observation of an absence of an exercise-induced increase in oxidative capacity (reflected by SDH OD) may be related to a differential response between muscles. In fact, in line with our observation, they observed no exercise-induced increase in the citrate synthase activity in the plantaris muscle. We therefore suggest that this protocol did not provide enough intensity or volume to induce angiogenesis and increases in oxidative capacity in the plantaris muscle.

### Effects of overload by synergist inactivation

Overload of the plantaris muscle through denervation of the gastrocnemius and soleus induced hypertrophy and in young-adult mice only, a proportional rise in maximal isometric tetanic force, similar to that seen previously (Ballak et al. 2016). The hypertrophy was in both age groups accompanied with angiogenesis, as reflected by the increase in C:F and LCFR. This adaptation is well documented in the literature (Tickle et al. 2020; Ballak et al. 2016) and illustrates the coupling between fiber size and capillary supply (Bosutti et al. 2015), where it has been shown that the overload-induced hypertrophy and angiogenesis even follow a similar time course (Plyley et al. 1998).

Based on the inverse relationship between FCSA and SDH OD (oxidative capacity), we expected the hypertrophy to be accompanied with a reduction in SDH OD. Yet, we found no significant decrease in SDH OD (van der Laarse et al. 1998), something also seen before during overload-induced hypertrophy (Ballak et al. 2016).

### Effects of endurance exercise on overload-induced hypertrophy

In the present study, we demonstrated that overload-induced hypertrophy was not impaired by endurance exercise, suggesting that there was no trade-off between the adaptations to each training modality used in the current study. Although the regular endurance exercise in our study did not induce an increase in SDH OD, previous studies have shown an amplified hypertrophy and an undiminished increase in SDH activity in rats subject to both treadmill running and synergist ablation (Gollnick et al. 1981; Riedy et al. 1985). Perhaps even more significant is that the hypertrophic response was not even attenuated when combined with chronic electrical stimulation that elicited a significant rise in SDH activity (Frischknecht and Vrbová 1991). This and the higher oxidative capacity of fibers than expected for their size in hypertrophied muscles (Ballak et al. 2016) challenge the concept that there is a trade-off between fiber size and oxidative capacity (Degens 2012; van der Laarse et al. 1998; van Wessel et al. 2010) and add to mounting evidence that acute cellular interference effects do not necessarily translate to impaired adaptations to exercise (Murach and Bagley 2016).

### Impact of overload on endurance exercise-induced increases in fatigue resistance

According to van Wessel et al. (2010), the inverse relationship between fiber size and oxidative capacity suggests that combining endurance and resistance exercise impairs adaptations to endurance exercise and attenuates the hypertrophic response to resistance training. Similar to the absence of attenuated hypertrophy during concomitant endurance exercise and overload discussed above, the endurance exercise-induced increase in fatigue resistance was not diminished by concomitant overload. The proportional increase in C:F and fiber size, as reflected by the maintained CD and CFD, during hypertrophy prevented an increase in the average diffusion distance from capillaries to mitochondria, and hence minimized any hypertrophy-associated diffusion limitations of oxygen to the working mitochondria (Degens 2012). Angiogenesis may thus explain a maintained but not an increased fatigue resistance. As discussed above, another factor is the type IIb to IIa fiber type transition that occurs irrespective of concomitant overload that will reduce the ATP demand during isometric contractions.

### Responses to overload and endurance exercise in old mice

The absence of blunted hypertrophy and fatigue resistance when both endurance exercise and overload were combined was seen in both young-adult and old mice. However, the increase in muscle mass in response to overload was attenuated in old mice, something seen also in “early ageing” mice (Ballak et al. 2016) and in response to loaded voluntary wheel running (Soffe et al. 2016). Some have even found no hypertrophy with overload at this age (Lee et al. 2016). The smaller increase in muscle mass in old animals is not due to differing proportions of connective tissue in the two age groups and it appeared that the percentage increase in FCSA was also similar between young and old. The blunted increase in muscle mass with overload in spite of similar fiber hypertrophy to that with young animals is comparable with other examples of discrepancies in measurement of hypertrophy noted elsewhere (Haun et al. 2019). Regardless of this, our data show the adaptive response to overload is diminished in old muscle.

Part of the diminished hypertrophic response may be attributable to a lower LCFR for type IIb fibers in old mice, as in elderly men with lower type II capillarization demonstrated less muscle fiber growth with resistance training than those with higher type II capillarization (Snijders et al. 2017). However, the overall capillary density of the muscle was even higher in old than young mice, and following the argument above, should lead to an enhanced rather than a diminished hypertrophic response. Here we propose like Ballak et al. (2016) that the impaired hypertrophic response is at least partly due to the impaired angiogenic response, as hypertrophy has a similar time course to capillary growth (Plyley et al. 1998; Egginton et al. 2011).

It has been reported previously in rats that at very advanced age (33 and 36 months) overload may not result in hypertrophy and even cause a reduction in the specific tension (Degens and Alway 2003; Blough and Linderman 2000). Combining our current data with previous data (Ballak et al. 2015), the pattern emerges that also in mice overload in early ageing is accompanied with a commensurate increase in force-generating capacity, while the specific tension is reduced with overload in older mice. Also in humans, some studies report a similar hypertrophy in older people and young-adults (Cannon et al. 2007; Kryger and Andersen 2007), but others report attenuated increases in thigh muscle cross-sectional area (Kraemer et al. 1999), attenuated fiber size (Slivka et al. 2008), or even no hypertrophy at all in very old (83–94 years old) people (Karlsen et al. 2019). Whatever the cause of these discrepant observations between human studies, it seems that the hypertrophic response deteriorates with age, where the muscle has potentially already maximally activated its regeneration potential (Edstrom and Ulfhake 2005), not being able to cope with any additional challenges. Therefore, chronic mechanical tension such as overload may be detrimental given the impaired muscle recovery response in old animals (Brooks and Faulkner 1990).

The LCFR divided by the SDH-INT (FCSA * SDH OD) gives an indication of the oxygen supply to demand ratio of a muscle fiber. Here we showed, as even seen in early ageing in previous studies (Hepple and Vogell 2004; Messa et al. 2019), that there was a capillary supply in excess of the oxygen demand in old age. The oxygen supply to demand ratio was, however, reduced with overload in old animals only as a consequence of the impaired angiogenesis. Nevertheless, fatigue resistance was still elevated after exercise even in the overloaded muscles. This again indicates that the muscle fatigue resistance in our test is probably more determined by the ATP demand than the aerobic ATP generating capability of the muscle fibers.

### Limitations

The endurance training may have been of too low volume and intensity to induce capillary growth and increases in oxidative capacity. As a consequence, the overload and endurance exercise stimuli may have been insufficient to challenge the trade-off between fiber size and oxidative capacity. Nevertheless, they do show that these types of exercise can be combined to gain the benefits of each exercise modality. In fact, the reduction in specific tension in the old mice after our overload stimulus may well be an indication that we were already close to the capability of the muscle to adapt to a new challenge, and there is danger that longer and more intense training stimuli would result in damage rather than gains in muscle function. The extent of hypertrophy induced by overload via denervation of synergists is much greater than that in response to resistance training. Rather than a weakness, this is a strength of the present study as the large hypertrophic response is more likely to reveal limits of any trade-off between fiber size and oxidative capacity than the hypertrophy during conventional resistance exercise. The stress associated with the use of a shock grid to encourage running may have attenuated any extra hypertrophy that could be induced by exercise (Conner et al. 2014), but this effect is likely limited as the hypertrophic response was not diminished with exercise. Although this study only used male mice and results may differ in females, this is unlikely as the trade-off between fiber size and oxidative capacity is a function of diffusion limitations, a physical property that is independent of sex.

## Conclusion

Our data indicate that combining endurance exercise and overload induces the benefits of both stimuli without diminishing the adaptations of either and that the smaller increases in muscle mass found in old mice may be due to impaired angiogenesis with overload. The muscles of older animals might have been at the limits of their adaptive capacity as indicated by the reduction in specific tension after overload.

## Electronic supplementary material

ESM 1 (DOCX 26 kb)
