# Supplementary material for: Regular endurance exercise of overloaded muscle of young and old male mice does not attenuate hypertrophy and improves fatigue resistance
Source: GeroScience. 2020 Jul 8;43(2):741–57. doi: 10.1007/s11357-020-00224-x (PMC8110681; doi:10.1007/s11357-020-00224-x)
Supplement: Supplementary file 1 — (DOCX 26 kb) [file 11357_2020_224_MOESM1_ESM.docx]

**Supplementary table 1**: Local capillary to fiber ratio (LCFR) and capillary fiber density (CFD) for different fiber types in the oxidative and glycolytic regions of plantaris muscles exposed to overload and endurance stimuli in young and old C57BL/6J mice.

|  | **Young** | | | | **Old** | | | | **Effects and interactions** |
| --- | --- | --- | --- | --- | --- | --- | --- | --- | --- |
|  | **SC** | **SO** | **EC** | **EO** | **SC** | **SO** | **EC** | **EO** |  |
| **Oxidative region** |  |  |  |  |  |  |  |  |  |
| Type IIa |  |  |  |  |  |  |  |  |  |
| LCFR | 1.06 ± 0.37 | 1.70 ± 0.39 | 0.93 ± 0.29 | 1.60 ± 0.31 | 1.20 ± 0.46 | 1.26 ± 0.17 | 1.16 ± 0.14 | 1.51 ± 0.33 | R***, H***, OH** |
| CFD | 1496 ± 196 | 1398 ± 479 | 1503 ± 502 | 1381 ± 238 | 1881 ± 429 | 1314 ± 268 | 2146 ± 163 | 1468 ± 299 | O**, R***, H***, OH** |
| Type IIa/IIx |  |  |  |  |  |  |  |  |  |
| LCFR | 1.44 ±  0.39 | 2.17 ±  0.64 | 1.34 ±  0.80 | 2.08 ±  0.69 | 1.60 ±  0.69 | 1.36 ±  0.28 | 1.61 ±  0.26 | 1.95 ±  0.64 | H* |
| CFD | 1481 ±  158 | 1246 ± 373 | 1225 ±  188 | 1294 ±  244 | 1792 ±  374 | 1113 ±  234 | 2025 ± 225 | 1453 ±  363 | O*, R***, H**, OR***, RH**, RE** |
| Type IIx |  |  |  |  |  |  |  |  |  |
| LCFR | 1.71 ± 0.58 | 2.33 ± 0.49 | 1.23 ± 0.33 | 2.56 ± 0.80 | 1.76 ± 0.57 | 1.84 ± 0.22 | 1.74 ± 0.34 | 1.86 ± 0.42 | R**, H***, OH*** |
| CFD | 1346 ± 254 | 1217 ± 466 | 1200 ± 405 | 1168 ± 239 | 1598 ± 449 | 1220 ± 386 | 1830 ± 247 | 1204 ± 205 | O*, R*, H** |
| Type IIb/IIx |  |  |  |  |  |  |  |  |  |
| LCFR | 1.59 ±  0.17 | 2.13 ±  0.46 | 1.43 ±  0.59 | 2.03 ±  0.36 | 1.62 ±  0.29 | 1.62 ±  0.28 | 1.62 ±  0.35 | 2.12 ±  0.29 | R*, H***, OH*, OE* |
| CFD | 1172 ±  294 | 956 ±  350 | 982 ±  446 | 987 ±  176 | 1578 ±  431 | 1112 ±  264 | 1440 ±  351 | 1359 ±  90 | O*, H***, RE* |
| Type IIb |  |  |  |  |  |  |  |  |  |
| LCFR | 1.76 ± 0.18 | 2.56 ± 0.32 | 1.50 ± 0.44 | 2.50 ± 0.55 | 1.81 ± 0.68 | 1.73 ± 0.18 | 1.80 ± 0.53 | 1.93 ± 0.35 | O*, R**, H***, OH*, OE* |
| CFD | 1098 ± 147 | 1033 ± 439 | 1013 ± 396 | 952 ± 197 | 1401 ± 371 | 1005 ± 259 | 1487 ± 169 | 995 ± 274 | O*, R***, H** |
| **Glycolytic region** |  |  |  |  |  |  |  |  |  |
| Type IIa |  |  |  |  |  |  |  |  |  |
| LCFR | 0.73 ± 0.12 | 1.56 ± 0.71 | 0.71 ± 0.30 | 1.13 ± 0.27 | 1.06 ± 0.20 | 1.06 ± 0.33 | 0.91 ± 0.18 | 0.93 ± 0.17 | R***, H***, OH** |
| CFD | 1150 ± 230 | 1105 ± 242 | 1229 ± 364 | 1236 ± 303 | 1769 ± 334 | 1324 ± 110 | 1880 ± 262 | 1278 ± 126 | O**, R***, H***, OH** |
| Type IIa/IIx |  |  |  |  |  |  |  |  |  |
| LCFR | 1.09 ±  0.31 | 1.77 ±  0.73 | 1.39 ±  1.15 | 1.46 ±  0.18 | 1.21 ±  0.54 | 1.67 ±  0.76 | 1.09 ±  0.20 | 1.56 ±  0.35 | H* |
| CFD | 1216 ± 73 | 1016 ±  199 | 1294 ±  244 | 1018 ±  124 | 1631 ±  438 | 1534 ±  410 | 1784 ±  89 | 1185 ±  127 | O*, R***, H**, OR***, RH**, RE** |
| Type IIx |  |  |  |  |  |  |  |  |  |
| LCFR | 1.09 ± 0.19 | 2.00 ± 0.53 | 1.05 ± 0.33 | 1.89 ± 0.52 | 1.42 ± 0.14 | 1.41 ± 0.34 | 1.34 ± 0.29 | 1.86 ± 0.42 | R**, H***, OH*** |
| CFD | 1062 ± 248 | 924 ± 244 | 1159 ± 239 | 1076 ± 302 | 1314 ± 403 | 1161 ± 278 | 1581 ± 155 | 1209 ± 155 | O*, R*, H** |
| Type IIb/IIx |  |  |  |  |  |  |  |  |  |
| LCFR | 1.26 ±  0.47 | 2.24 ±  0.41 | 1.04 ±  0.51 | 1.62 ±  0.56 | 1.46 ±  0.24 | 1.79 ±  0.77 | 1.37 ±  0.47 | 1.64 ±  0.43 | R*, H***, OH*, OE* |
| CFD | 989 ±  189 | 768 ±  131 | 991 ±  553 | 830 ±  212 | 1445 ±  521 | 1123 ±322 | 1221 ±  222 | 1052 ±  181 | O*, H***, RE* |
| Type IIb |  |  |  |  |  |  |  |  |  |
| LCFR | 1.50 ± 0.24 | 2.53 ± 0.30 | 1.29 ± 2.5 | 1.70 ± 0.46 | 1.41 ± 0.16 | 1.81 ± 0.53 | 1.37 ± 0.28 | 1.92 ± 0.21 | O*, R**, H***, OH*, OE* |
| CFD | 899 ± 137 | 813 ± 170 | 879 ± 323 | 836 ± 247 | 1201 ± 446 | 1043 ± 186 | 1245 ± 140 | 997 ±  76 | O*, R***, H** |

SC = sedentary control, SO = sedentary overload, EC = exercise control, EO = exercise overload. Data are expressed as means ± standard deviation. To signify significant differences: O= old age effect, R=region effect, H=hypertrophy effect, E=exercise effect. To signify interactions: OR=old age*region interaction, OH= old age*hypertrophy interaction, OR=old age*region interaction, OE=old age*exercise interaction, RH=region*hypertrophy interaction, RE= region*exercise interaction. To denote statistical significance: *denotes p<0.05, ** denotes p<0.01, ***denotes p<0.001.

**Supplementary table 2:** Succinate dehydrogenase optical density (SDH OD) for different fiber types in the oxidative and glycolytic regions of plantaris muscles exposed to overload and endurance training in young and old C57BL/6J mice.

|  | **Young** | | | | **Old** | | | | **Effects and interactions** |
| --- | --- | --- | --- | --- | --- | --- | --- | --- | --- |
|  | **SC** | **SO** | **EC** | **EO** | **SC** | **SO** | **EC** | **EO** |  |
| **SDH OD** |  |  |  |  |  |  |  |  |  |
| **Deep** |  |  |  |  |  |  |  |  |  |
| Type I | - | 0.196  ±  0.038 | 0.320  ±  0.000 | 0.232  ±  0.054 | 0.131  ±  0.000 | - | 0.156  ±  0.028 | 0.192  ±  0.000 | - |
| Type I/IIa | - | 0.2182 ±  0.061 | - | - | - | 0.160  ±  0.000 | - | - | - |
| Type IIa | 0.284  ± 0.092 | 0.256  ± 0.073 | 0.413  ±  0.146 | 0.320  ±  0.099 | 0.232  ±  0.103 | 0.188  ±  0.055 | 0.236  ±  0.045 | 0.208  ± 0.051 | O**, H** |
| Type IIa/IIx | 0.300  ±  0.140 | 0.231  ±  0.081 | 0.407  ±  0.201 | 0.251  ±  0.087 | 0.209  ±  0.058 | 0.178  ± 0.056 | 0.186  ±  0.02 | 0.145  ±  0.011 | O*, H**, OH**, OE** |
| Type IIx | 0.203  ±  0.052 | 0.195  ±  0.074 | 0.290  ± 0.089 | 0.230  ±  0.077 | 0.178  ±  0.055 | 0.170  ±  0.062 | 0.160  ±  0.043 | 0.152  ±  0.069 | O* |
| Type IIb/IIx | 0.166  ±  0.054 | 0.155  ±  0.062 | 0.269  ±  0.173 | 0.161  ±  0.049 | 0.151  ±  0.055 | 0.140  ±  0.028 | 0.129  ± 0.008 | 0.118  ±  0.011 | O*, RH*, RE** |
| Type IIb | 0.122  ± 0.028 | 0.107  ± 0.020 | 0.139  ±  0.029 | 0.117  ± 0.033 | 0.113  ±  0.035 | 0.125  ± 0.045 | 0.112  ± 0.015 | 0.109  ± 0.011 |  |
| **Superficial** |  |  |  |  |  |  |  |  |  |
| Type I | - | 0.205  ±  0.05 | 0.140  ±  0.080 | 0.337  ±  0.000 | - | - | - | - | - |
| Type I/IIa | - | 0.318  ±  0.115 | - | - | - | - | - | - | - |
| Type IIa | 0.300  ± 0.107 | 0.258  ±  0.058 | 0.385  ±  0.104 | 0.319  ± 0.118 | 0.210  ±  0.088 | 0.196  ±  0.065 | 0.218  ± 0.045 | 0.239  ±  0.058 | O**, H** |
| Type IIa/IIx | 0.260  ±  0.140 | 0.249  ±  0.079 | 0.385  ±  0.148 | 0.252  ± 0.091 | 0.193  ±  0.114 | 0.220  ±  0.124 | 0.158  ± 0.027 | 0.163  ±  0.022 | O*, H**, OH**, OE** |
| Type IIx | 0.2712  ± 0.124 | 0.191  ±  0.060 | 0.275  ±  0.091 | 0.244  ±  0.105 | 0.179  ±  0.053 | 0.183  ±  0.097 | 0.144  ±  0.018 | 0.170  ± 0.062 | O* |
| Type IIb/IIx | 0.209  ±  0.077 | 0.135  ±  0.029 | 0.222  ±  0.194 | 0.171  ± 0.057 | 0.134  ±  0.032 | 0.152  ±  0.060 | 0.115  ± 0.011 | 0.124  ±  0.008 | O*, RH*, RE** |
| Type IIb | 0.128  ±  0.038 | 0.111  ±  0.012 | 0.122  ±  0.022 | 0.122  ±  0.034 | 0.104  ±  0.033 | 0.125  ±  0.054 | 0.109  ±  0.020 | 0.104  ±  0.027 |  |

SC = sedentary control, SO = sedentary overload, EC = exercise control, EO = exercise overload. Data are expressed as means ± standard deviation. To signify significant differences: O= old age effect, R=region effect, H=hypertrophy effect, E=exercise effect. To signify interactions: OR=old age*region interaction, OH= old age*hypertrophy interaction, OR=old age*region interaction, OE=old age*exercise interaction, RH=region*hypertrophy interaction, RE= region*exercise interaction. To denote statistical significance: *denotes p<0.05, ** denotes p<0.01, ***denotes p<0.001.
